# Polarity-specific high-level information propagation in neural networks

**DOI:** 10.3389/fninf.2014.00027

**Published:** 2014-03-17

**Authors:** Yen-Nan Lin, Po-Yen Chang, Pao-Yueh Hsiao, Chung-Chuan Lo

**Affiliations:** ^1^Institute of Systems Neuroscience, National Tsing Hua UniversityHsinchu, Taiwan; ^2^Department of Life Science, National Tsing Hua UniversityHsinchu, Taiwan

**Keywords:** neural networks, *Drosophila*, *Caenorhabditis elegans*, central complex, polarity, information propagation

## Abstract

Analyzing the connectome of a nervous system provides valuable information about the functions of its subsystems. Although much has been learned about the architectures of neural networks in various organisms by applying analytical tools developed for general networks, two distinct and functionally important properties of neural networks are often overlooked. First, neural networks are endowed with polarity at the circuit level: Information enters a neural network at input neurons, propagates through interneurons, and leaves via output neurons. Second, many functions of nervous systems are implemented by signal propagation through high-level pathways involving multiple and often recurrent connections rather than by the shortest paths between nodes. In the present study, we analyzed two neural networks: the somatic nervous system of *Caenorhabditis elegans* (*C. elegans*) and the partial central complex network of *Drosophila*, in light of these properties. Specifically, we quantified high-level propagation in the vertical and horizontal directions: the former characterizes how signals propagate from specific input nodes to specific output nodes and the latter characterizes how a signal from a specific input node is shared by all output nodes. We found that the two neural networks are characterized by very efficient vertical and horizontal propagation. In comparison, classic small-world networks show a trade-off between vertical and horizontal propagation; increasing the rewiring probability improves the efficiency of horizontal propagation but worsens the efficiency of vertical propagation. Our result provides insights into how the complex functions of natural neural networks may arise from a design that allows them to efficiently transform and combine input signals.

## Introduction

Nervous systems are characterized by their ability to receive tremendous amounts of information, to process it in parallel, and to produce complex responses. Even in small animals such as *Caenorhabditis elegans* (*C. elegans*) and *Drosophila*, the nervous systems are extremely complex and are still under intensive investigation (Chiang et al., 2011; Varshney et al., 2011). In recent years, due to technological advances in identifying and labeling single neurons, we have begun to be able to reconstruct the connectome at the neuronal and synaptic levels. In response to the availability of the new data, various statistical and mathematical tools have been applied to analyzing connectomes, including those of *C. elegans*, retinal networks, monkey cortical networks, functional networks of human brains, etc. (Bullmore and Sporns, 2009; Sporns, 2010, 2011; Briggman et al., 2011; Varshney et al., 2011). Most of these tools were developed for general networks and focused on properties that can be divided into three categories (Sporns, 2010; Rubinov and Sporns, 2010): (1) local segregation concerning clustering and modularity (Watts and Strogatz, 1998; Girvan and Newman, 2002; Guimerà and Amaral, 2005; Leicht and Newman, 2008), (2) global integration concerning degree distribution, characteristic path length and efficiency (Watts and Strogatz, 1998; Amaral et al., 2000; Latora and Marchiori, 2001; Estrada and Hatano, 2008), and (3) the influence and centrality related to “hubs,” which significantly alter the connectivity of a network (Freeman, 1979; Guimerà and Amaral, 2005).

However, two critical aspects of neural networks are not considered in most analyses. First, neural networks are endowed with polarity that defines a global direction of information flow. Specifically, neural networks are often characterized by neurons receiving external input from the environment or from other brain regions, neurons sending information out of the network to other brain regions or to the motor systems, and neurons that only make contacts within the network. Second, direct links or shortest pathways may not be functionally more important than the higher level pathways, in which signals travel through multiple, and often recurrent, connections. Let us take the oculomotor system of mammals as an example. A suddenly appearing visual stimulus can trigger a reflexive rapid eye movement toward the stimulus via the retinotectal pathway, which acts as a shortcut from the retina to the eye-movement-command neurons of the superior colliculus (Sparks, 2002; Munoz and Everling, 2004). On the other hand, visual signals can also elicit voluntary eye movements by propagating through long and complex pathways that involve recurrent networks comprising visual cortex, parietal cortex, frontal cortex and basal ganglia (Munoz and Everling, 2004). These voluntary movements are typically associated with high-level cognitive functions such as object recognition. Although being much slower than the reflexive responses, these cognitive functions are still incredibly efficient. Think about that we can recognize an object within hundreds of milliseconds while such a process involves no less than hundreds of millions of neurons.

**Figure 1:**
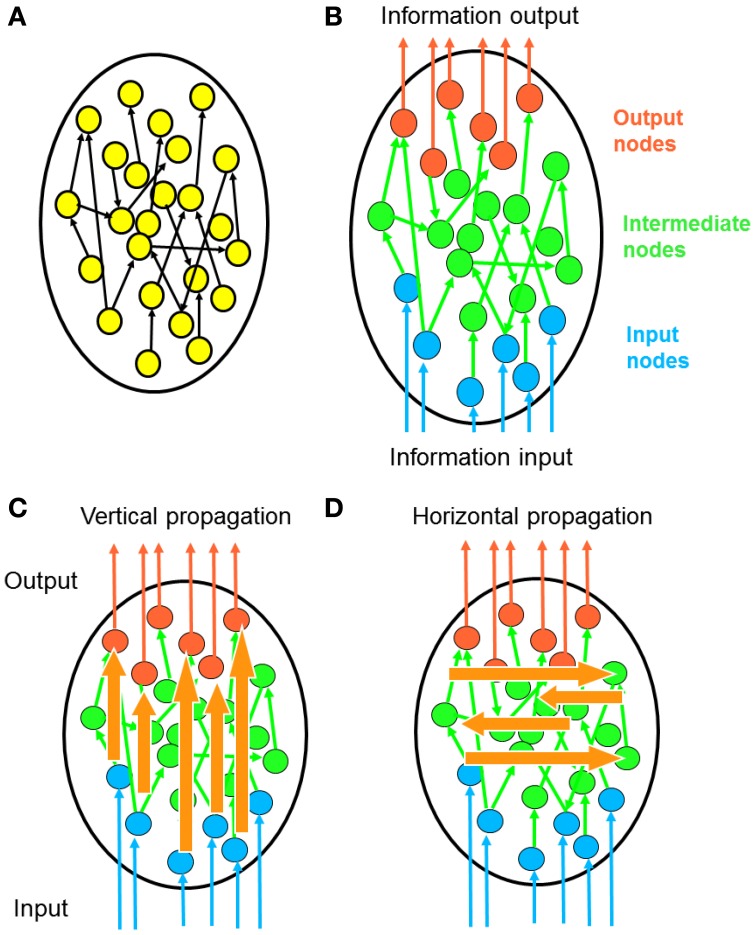
**Polarity of network information propagation. (A)** In a typical network, information propagates within the network and no specific entry or leaving point is specified. **(B)** In many neural networks, signals enter the network from a specific set of input neurons and leave the network via a specific set of output neurons. This global direction of information flow called the “polarity” of a neural network. **(C)** Considering that polarity is a fundamental property of neural networks, it is meaningful to investigate how information propagates along the direction of polarization. Each signal from an input neuron undergoes transformation by complex propagation pathways before leaving at an output neuron. We term this process vertical propagation. **(D)** We can also investigate how information propagates across the polarization direction. Through such horizontal propagation, each input signal may contribute information to a large number of output nodes. As a result, input signals combine with each other and new information is generated.

Based on the foregoing statements, we argue that although we can quantify the efficiency of a neural network by examining the direct connections between every node, what really matters is how efficiently information propagates from input to output nodes, and not just through the shortest pathways, but also through pathways involving multiple intermediate nodes. Following this line of thought, we here propose a novel method of network architecture analysis for quantifying the efficiency of information propagation along different directions with respect to the network polarity (Figure 1). Using our method, we showed that two natural neural networks significantly outperform artificially generated networks in information propagation.

## Methods

In the present study, we analyzed information propagation in neural networks based on their connection matrices. We defined the connection matrix, also known as the adjacency matrix, *A*(*i, j*), as the one-way connectivity from node (or neuron) *i* to node *j*. Each element in the matrix can take on a value of either 0 (no connection) or 1 (one connection). We analyzed several neural networks and artificial networks, including the chemical synaptic network of the *C. elegans* hermaphrodite somatic nervous system (CE) (Varshney et al., 2011), the partial central complex neural network of *Drosophila* (CX), classic Watts-Strogatz small-world (SW) networks (Watts and Strogatz, 1998), ring lattice regular (RL) networks (Watts and Strogatz, 1998), and Erdős–Rényi random (ER) networks (Erdős and Rényi, 1960).

### *C. elegans* (CE) network

The nervous system of the *C. elegans* hermaphrodite consists of 302 neurons, with 282 in the somatic nervous system and the remaining 20 in the pharyngeal nervous system. In the present study, we used the recently updated somatic neural network of *C. elegans* (Varshney et al., 2011), which is based on a combination of multiple datasets (White et al., 1986; Durbin, 1987; Hall and Russell, 1991). We deleted three neurons (CANL/R, VC06) with no known connections and then formed an updated network comprising 279 somatic neurons and 2194 synaptic connections.

### Partial central complex (CX) network of *Drosophila*

The data on the partial central complex network of *Drosophila* were derived from a recently published study (Lin et al., 2013) that described the networks formed by neurons innervating the protocerebral bridge, a major neuropil of the central complex. The network consists of 194 neuronal types, each with a unique innervation pattern. See Supplementary Method for detailed descriptions of the dataset.

### Watts-Strogatz small-world (SW)

Small-world networks are characterized by a clustering coefficient *C* and a characteristic path length *L* (Watts and Strogatz, 1998). In the present study, we used the directed version of characteristic path length (Rubinov and Sporns, 2010) and clustering coefficient (Fagiolo, 2007) that are implemented in the brain connectivity toolbox (Rubinov and Sporns, 2010).

To enable comparisons with the natural neural networks, we constructed directed SW networks based on the classical Watts-Strogatz one-dimensional ring lattice with 297 nodes and 2194 connections as in the CE network. Unless otherwise mentioned, we set the rewiring probability of the SW network to 0.3 because the resulting characteristic path length *L* (Watts and Strogatz, 1998; Rubinov and Sporns, 2010), clustering coefficient *C* (Watts and Strogatz, 1998; Fagiolo, 2007; Rubinov and Sporns, 2010) and small-worldness *S* (Humphries and Gurney, 2008) of the SW network were similar to those of the CE network (*L*_*CE*_ = 3.44, *C*_*CE*_ = 0.21, *S*_*CE*_ = 6.42; *L*_*SW*_ = 3.20, *C*_*SW*_ = 0.23, *S*_*SW*_ = 7.60).

The small-worldness *S* of a network is defined as the ratio of its *C* and *L* normalized by that of the Erdős–Rényi random (ER) network (described below):

$$S=\frac{C/L}{C_{ER}/L_{ER}}.$$

### Regular lattice (RL)

The RL networks were constructed by building SW networks as stated above using a rewiring probability *p* = 0.

### Erdős–Rényi random (ER) networks

Random networks were generated using the Erdős–Rényi model (Erdős and Rényi, 1960) with self-loops forbidden. To facilitate comparison with the CE network, the ER networks contained 279 nodes, and the connection probability was set to 0.0283, which leads to the desired CE connection number of 2194.

### Selection of input and output nodes

In the present study, we investigated how information propagates through networks in which fixed input nodes and output nodes form the entry points and leaving points, respectively, for information. For the CE network, because it represents the somatic nervous system of the entire organism, we selected the 88 sensory neurons as the input nodes and the 109 motor neurons as the output nodes. The remaining 82 neurons were classified as interneurons/internodes. To facilitate comparison with the CE network, we randomly selected 88 nodes as input nodes, 82 nodes as internodes, and 109 nodes as output nodes in each of the RL, SW, and ER networks. In the partial CX network, the input neurons were defined as neurons which project their dendrites to (i.e., receive information from) the neuropils CCP, CVLP, and VMP, which convey visual information to the central complex (Lin et al., 2013). The output neurons were defined as neurons which project their axons to (i.e., send signals to) the neuropil IDFP, which directs signals from the central complex to the locomotor circuits (Lin et al., 2013).

To see the influence of polarity on information propagation in a network, we varied the network polarity by varying the spatial relationships between input and output nodes in the SW network. Instead of selecting the input and output nodes randomly, we first selected the input nodes one next to another on the ring so that they formed a large and continuous cluster that accounted for roughly 1/3 of the ring. Next, we selected output nodes the same way but placing them on the opposite side of the ring. The input and output node clusters were separated by two internode clusters, each accounting for roughly 1/6 of the ring.

In addition, we tested how artificially reassigning the input and output neurons in the CE network changes its vertical and horizontal propagation characteristics. We tested three different ways of selecting input and output neurons:

1. Rand I/O: Neuron type (input, output, or inter) was randomly assigned but the number of neurons belonging to each type was held the same as in the original CE network.
2. Separated I/O: Input and output neurons were randomly assigned but the two neuron types were confined to different modules so that each module contained only input + inter or only output + inter neurons. The number of neurons belonging to each type was also held the same as in the original CE network.
3. Reversed I/O: Input and output neuron types were interchanged relative to the original CE network, giving 109 input nodes, 82 internodes, and 88 output nodes.

The modules of the CE network were identified for purposes of assignment method (2) using the algorithm proposed in (Leicht and Newman, 2008; Rubinov and Sporns, 2010).

### Channel connectivity matrix

**Figure 2:**
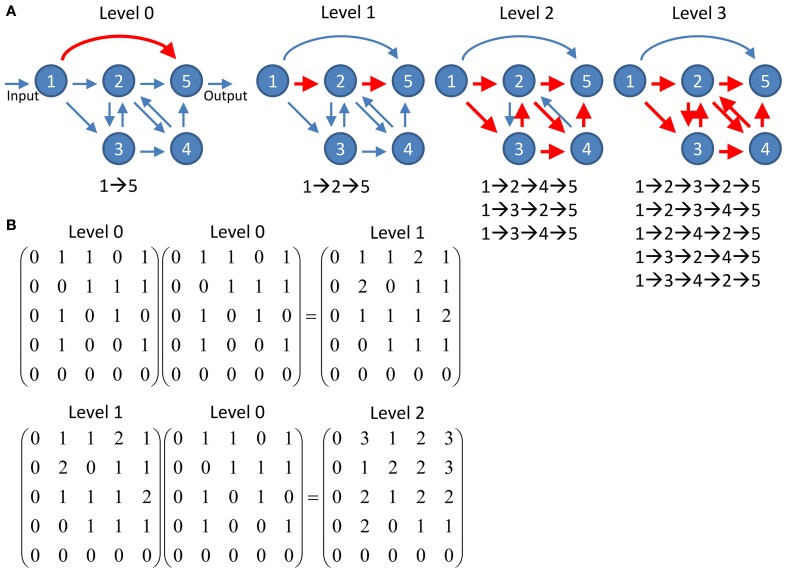
**Schematics of information propagation at different levels. (A)** An example network of five nodes. There is one pathway (direction connection between the input node and the output node) at propagation level 0 and one pathway (nodes 1→2→5) at level 1. The number of pathways quickly increases to 3 and 5 at levels 2 and 3, respectively. Note that the feedforward direct connection between node 1 and 5 at level 0 does not involve in any pathway at higher levels while the increasing number of pathways at higher levels is contributed by the recurrent circuit comprises nodes 2, 3, and 5. **(B)** The number of pathways connecting any two nodes at given propagation level can be computed by simple matrix multiplication. The level 0 connectivity matrix represents the adjacency matrix of the network shown in **(A)**. The connectivity matrix at any given level *n* can be computed by multiplication of the matrix at level *n* - 1 and the matrix at level 0. The value of each element in the matrices indicates the number of pathways connecting the two corresponding nodes.

To quantify information propagation from input nodes to output nodes through multiple connections, we studied the networks at different propagation levels. For given input and output nodes, they often can be connected via various pathways which involve different number of intermediate nodes. The propagation level *l* is defined as the number of intermediate nodes making up a pathway between given input and output nodes (Figure 2). For example, a pathway I→A→B→O which connects the input node I and the output node O via intermediate nodes A and B is a level 2 pathway. Note that recurrent pathways which pass through the same nodes multiple times also count. Therefore the pathway I→A→B→A→B→O is counted as a level 4 pathway. We calculated the number of pathways between each input and output node at various propagation levels.

We present the number of pathways at a given propagation level using the channel connectivity matrices. Assuming a network with *n*_*in*_ input nodes and *n*_*out*_ output nodes, there are *n*_*in*_ × *n_out_* potential channels of information propagation formed by pairing input and output nodes in all possible ways. We define an *n*_*in*_ × *n_out_* channel connectivity matrix as:

$$M{\left(i,j\right)}_{l}=\log_{10}\left(m{\left(i,j\right)}_{l}+b\right),$$

where *m*(*i, j*)*_l_* is a matrix representing the number of pathways from node *i* to *j* at the propagation level *l*. We call a channel between nodes *i* and *j* “connected” at the level *l* if *m*(*i, j*)_*l*_ ≠ 0 while we call the channel “not connected” or “disconnected” if *m*(*i, j*)_*l*_ = 0. In highly recurrent networks such as a typical neural circuit, the values of *m*(*i, j*)*_l_* grow exponentially with an exponent of *l*. To prevent highly recurrent channels from completely dominating the results in our subsequent analysis, we take the logarithm of *m*(*i, j*)*_l_*. (*b* = 0.1) is a small value added to prevent the occurrence of log _10_ 0, which cannot be computed. The matrix *m*(*i, j*)*_l_* is easily constructed using the adjacency matrix, *A*(*i, j*), based on the fact that the number of possible *k*-step pathways starting at node *i* and terminating at node *j* is given by *A*^*k*^(*i, j*) (Biggs, 1993). Therefore, *m*(*i, j*)_*l*_ can be extracted from *A*^*l* + 1^(*i, j*).

We observed that among the lower propagation levels, many new channels are created at each increase in level, resulting in a distinct pattern of channel connectivity at each level. With increasing level, although number of pathways per connected channel still increases with an exponent of *l*, strongly connected channels, i.e., channels with a large number of pathways, remain strongly connected and weakly connected channels remain relatively weak. In consequence, the connectivity pattern stabilizes.

### Vertical and horizontal propagation

Using the channel connectivity matrices, we studied how information propagates vertically (along the direction of polarization) and horizontally (across the direction of polarization) in the networks (Figure 1).

The varying pattern of channel connectivity at low propagation levels indicates the evolution of information propagation across levels, while a stabilized connectivity pattern at high levels indicates the establishment of a persistent pattern of information propagation in the network. Therefore, we can measure the efficiency of information propagation between input and output nodes by examining how quickly the channel activity matrix approaches its final pattern. To this end, we defined the degree of vertical propagation, which measures the similarity between the matrices of consecutive propagation levels *l* and *l* + 1:

$$V_{l}=\underset{i,j}{\text{Corr}}\left(M{\left(i,j\right)}_{l},M{\left(i,j\right)}_{l+1}\right),$$

where $\underset{i,j}{Corr}()$ is the Pearson's correlation coefficient computed over elements specified by *i* and *j*. We note that in a recurrent network, the value of *M*(*i, j*) for each connected channel *i* − *j* increases indefinitely with propagation level due to the recurrent pathways. However, even that the *M*(*i, j*)'s keep increasing, *V_l_* still approaches 1 after a certain level as long as there is no large increase or decrease in the number of connected channels.

The degree of horizontal propagation measures how quickly signals entering an input node spread to different output nodes. Specifically, for each input node, we compute the index *h*(*i*)*_l_*, which is defined as the percentage of output nodes that have been connected to the input node *i* at a given level *l*. This is done by counting the percentage of non-zero *m*(*i, j*)*_l_*'s across all *j* for a given *i* and *l*. The average of this percentage over all input nodes, $H_{l}=\frac{1}{n_{in}}\sum_{i}h{\left(i\right)}_{l}$, gives the degree of horizontal propagation. A large *H_l_* means that each input signal is propagated to a large proportion of the output nodes, indicating strong horizontal propagation. From the output point of view, each output node receives converging signals from a large portion of input nodes when *H_l_* is large.

### Classification of hubs

In the present study, we investigated how hubs, or highly connected nodes, influence the vertical and horizontal propagations. Hubs can be classified into three classes: provincial, connector and kinless, based on the participation coefficient *P*_*i*_ which quantifies the distribution of the links of a hub among different modules in a network (Guimerà and Amaral, 2005). Therefore, to identify the type of a hub, we first need to identify modules in a network. We used the brain connectivity toolbox (Rubinov and Sporns, 2010) to identify modules in directed networks (Leicht and Newman, 2008).

After modules in a network were identified, we calculated the participation coefficient for 15 mostly connected nodes in the CE networks. The participation coefficient *P*_*i*_ of a node *i* is originally defined as

$$P_{i}=1-\sum_{s}^{N_{m}}{\left(\frac{k_{i,s}}{k_{i}}\right)}^{2},$$

where *N*_*m*_ is the number of modules, *k*_*i*_ is the degree of node *i*, and *k*_*i, s*_ is the number of links of node *i* in module *s*. In large*N*_*m*_ limit, *P*_*i*_ ≈ 1 indicates the uniformly distributed links of node *i* among all modules whereas *P*_*i*_ = 0 indicates the exclusive distribution of links in only one module. The three classes of hubs is defined as (1) provincial: *P* ≤ 0.30 (2) connector: 0.30 < *P* ≤ 0.75 and (3) kinless: *P* > 0.75 (Guimerà and Amaral, 2005).

However, the numbers of modules are small in some neural networks, such as the CE networks, which yield participation coefficients that are significantly smaller than 1 even for hubs that project their connections uniformly to all modules (Sporns et al., 2007). To address the issue, we slightly modified the definition for the participation coefficient by including a normalizing term:

$$P_{i}=\{\begin{array}{l} \left(1-\sum_{s}^{N_{m}}{\left(\frac{k_{i,s}}{k_{i}}\right)}^{2}\right)\times \left(\frac{N_{m}}{N_{m}-1}\right)\text{ when }N_{m}>1\\ 0\text{ when }N_{m}=1 \end{array}$$

Using the modified equation, the participation coefficient equals one for hubs with uniformly distributed links even in networks with a small number of modules.

### Computer scripts and data availability

The computer scripts and data used to generate the results presented in this paper are available for downloaded at http://life.nthu.edu.tw/~lablcc/codes/network_efficiency.html. The scripts were written and tested in Matlab R2012a.

## Results

**Figure 3:**
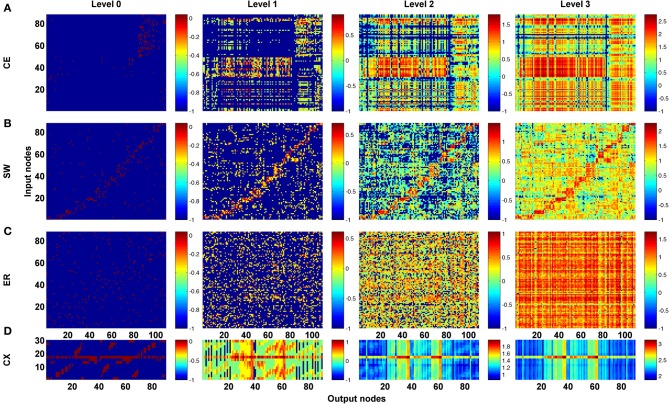
**The channel connectivity matrices demonstrate the existence of distinct information propagation patterns among different networks**. Each element in a matrix represents a pair made up of an input node/neuron and an output node/neuron (called a “channel”). The color of each element indicates the number (on a logarithmic scale, see Methods) of one-way paths between the given input and output nodes/neurons at the given propagation level. Therefore, the matrices display the connectivity of each channel. **(A)** The channel connectivity matrices at different levels for the *C. elegans* (CE) neural network. There is a significant difference in the channel connectivity between level 0 (direct link) and level 1 (through 1 intermediate node). However, the connectivity patterns are very similar between level 1 and level 2 (and higher), indicating that information propagation patterns are quickly stabilized above level 1 propagation. **(B)** The matrices for a classic small-world (SW) network. The connectivity patterns are more similar between level 0 and level 1 in SW compared to the CE network. **(C)** An Erdős–Rényi random (ER) network showing how the connectivity patterns are significantly different between each level and the next-higher level. **(D)** The partial central complex network (CX) of *Drosophila* demonstrating quickly stabilized channel connectivity patterns after level 1 as in the CE network.

As a first step, we computed the channel connectivity matrices for the *C. elegans* somatic neural network (CE), the partial central complex neural network of *Drosophila* (CX), the classic Watts-Strogatz small-world (SW) networks, the ring lattice regular (RL) networks, and the Erdős–Rényi random (ER) networks for different propagation levels (Figure 3). To help visualize the matrix representation, nodes were sorted using the algorithm described in Leicht and Newman (2008); Rubinov and Sporns (2010) so that nodes in tightly connected modules were placed together in the matrix. We found that the four types of network are characterized by distinct patterns in the channel connectivity matrices at propagation level 0, as expected due to different network architectures. Interestingly, as the propagation level increased, the matrix patterns developed differently among the four types. We made several observations:

1. The level 0 matrix, *M_0_*, of an SW network is characterized by the presence of a diagonal that represents highly connected local clusters. The diagonal persists at all levels, making the connectivity patterns visually similar to each other. As the level increases, more and more channels become connected, mainly through long-range projections.
2. The matrix of the ER network is characterized by randomly distributed dots with a pattern that changes significantly across each propagation level. Therefore, the channel connectivity matrices do not look similar between consecutive levels.
3. The matrices of the two neural networks, CE and CX, display patchy patterns at level 0. Interestingly, the patterns change dramatically at level 1 and then remain relatively stable at higher levels. This indicates that, although some input and output nodes are directly connected (level 0 connections), with increasing level, these connectivity patterns are quickly replaced by distinct patterns formed by multi-node, and often recurrent, connections. The involvement of recurrent pathways can be identified by checking the number of new nodes included in the pathways in each level. We will address this issue later.

**Figure 4:**
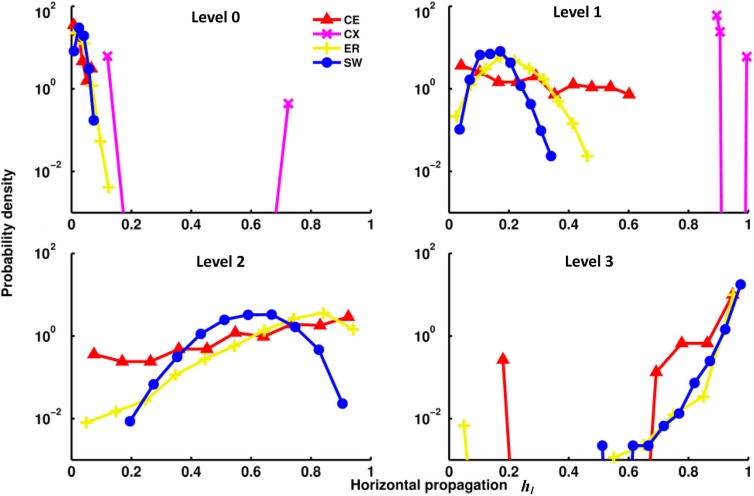
**Distributions of the horizontal propagation indexes *h_l_*(***i***) at different propagation levels**. There are significant differences in horizontal information propagation among different types of networks. Leftward-distributed indices *h* indicate that on average each input signal propagates to a larger proportion of output nodes than those in a network with rightward-distributed indices. The plots show that as the propagation level increases, the CX network has the fastest-growing horizontal propagation of all the networks. CE and ER have similar rates of increase of horizontal propagation while the SW network has the slowest.

The channel connectivity matrices indicate at which level a particular channel (a pair of input-output nodes) becomes connected and with how many pathways. Therefore, the matrices can be used to quantify vertical propagation. We will show our vertical propagation results later after we examine some of the properties of horizontal information propagation. To quantify horizontal propagation, we first computed the index *h*(*i*)*_l_* for each input node and then plotted the distribution of *h*(*i*)*_l_* (Figure 4). We observed that the *h*(*i*)*_l_*'s are distributed below 0.2 at level 0 for most networks, indicating that there is only a limited number of direct links from input to output nodes. However, as level increases, the distributions of the two neural networks (CE and CX) move toward 1 rapidly. Among the two artificial networks, ER and SW, ER performs as well as CE, but the distribution of *h*(*i*)*_l_* of SW changes much more slowly than that of the other networks, indicating that on average signals originating from a given input node of the SW network propagate to fewer output nodes at levels 1 and 2.

**Figure 5:**
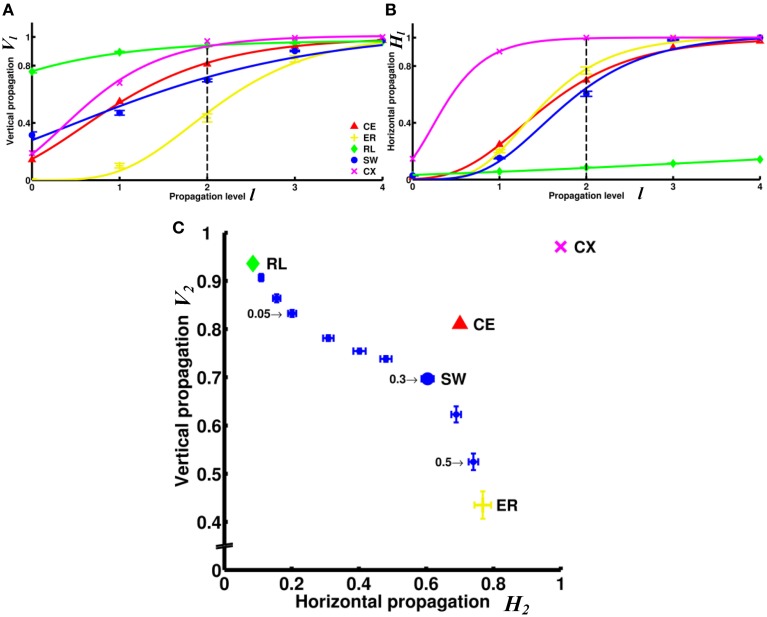
**Comparison of information propagation among different networks revealed that the two neural networks (CE and CX) have better overall horizontal and vertical propagation than other types of networks. (A)** The degree of vertical propagation *V_l_* as a function of the propagation level *l* across networks shows that while CE and CX networks start with relatively lower degrees of vertical propagation than RL and SW networks, the degrees grow faster than in other networks. **(B)** The degrees of horizontal propagation *H_l_* as a function of the propagation level show that CE, CX, and ER have relatively good horizontal propagation while the RL is the worst. To visualize the trend of the degrees of information propagation, we here fit the data with a Gompertz function. **(C)** Based on data shown in panels A and B, we represent the characteristics of each network by constructing a V_2_ - H_2_ plot. SW networks with different rewiring probability (selectively indicated by numbers) are shown for comparison. The two neural networks, CE and CX, are located near the upper right corner that characterizes high efficiency in both vertical and horizontal information propagation. Error bars indicate standard deviation calculated from 100 realizations.

The differences among the networks can be further examined by plotting degrees of vertical (*V_l_*) and horizontal (*H_l_*) propagation as functions of the propagation level *l* (Figures 5A,B). As a general trend, both degree of vertical and degree of horizontal propagation increase with propagation level. At level 4, both degrees in all networks (except the RL network) nearly reach the maximum value of 1. However, these networks are significantly different at lower propagation levels. The vertical propagation of the two neural networks CE and CX start from lower degrees at level 0, but climb relatively rapidly with increasing level. At level 2, midway between level 0 and level 4, the two neural networks have degrees of vertical propagation higher than those of artificial networks (except the RL networks). Interestingly, when we consider the degree of horizontal propagation at level 2 among the artificial networks, the RL network, which is very high in vertical propagation, becomes extremely low. In contrast, the ER network, which is low in vertical propagation, is now relatively high. The SW network remains at intermediate degrees on both propagation measures. At this level, the two neural networks CE and CX show high degrees on both measures.

The superiority of the two neural networks in terms of vertical and horizontal propagation can clearly be demonstrated by making a *V_2_* - *H_2_* plot (Figure 5C). We chose to use level 2 propagation (*V_2_* and *H_2_*) to construct the plot based on the following considerations: ideally, we want to compare the propagation efficiency at levels as high as possible because the goal here is to examine information propagation via pathways that involve multiple connections. However, the degrees of propagation in both directions are close to 1 at levels 3 and 4 for most networks and level 2 is the highest level at which we still observed distinct differences between networks. For comparison, we included small-world networks of various rewiring probability. The regular network is characterized by a very high degree of vertical propagation and an extremely low degree of horizontal propagation and is therefore situated at the upper left corner of the plot. As the rewiring probability increases, the networks exhibit progressively stronger small worldness, which leads to an increasing degree of horizontal propagation but a decreasing degree of vertical propagation. Consequently, the networks gradually move toward the lower left corner until they become completely random. This result indicates that there is a trade-off between vertical and horizontal propagation for the classic small-world networks, where we can obtain a high degree of vertical propagation or a high degree of horizontal propagation, but not both. Interestingly, we found that the two neural networks CE and CX are located away from the diagonal line on the plot formed by small-world networks and the two neural networks are characterized by high degrees of both vertical and horizontal propagation.

**Figure 6:**
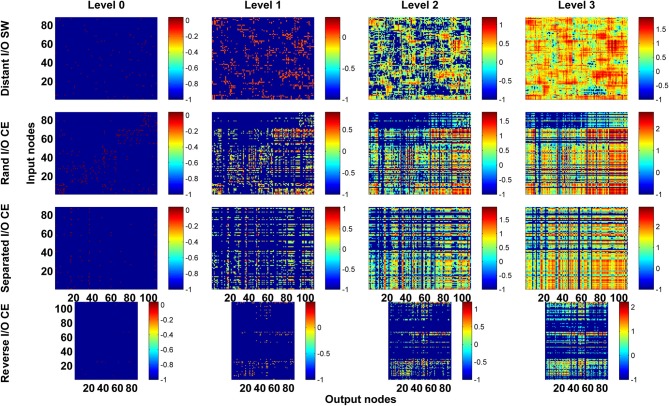
**Channel connectivity matrices showing that for the same network architecture, the pattern of information propagation can be significantly altered by changes in network polarity**. We test the SW network by reassigning input and output nodes to place them on opposite sides of the ring (Distant I/O SW). For the CE network, we test three different arrangements of input and output neurons. In Rand I/O CE, we randomly select neurons as input nodes or output nodes. In Separated I/O CE, we assign input and output neurons in such a way that input neurons are in different modules from output neurons. In reversed I/O CE, we interchange the input and output nodes.

**Figure 7:**
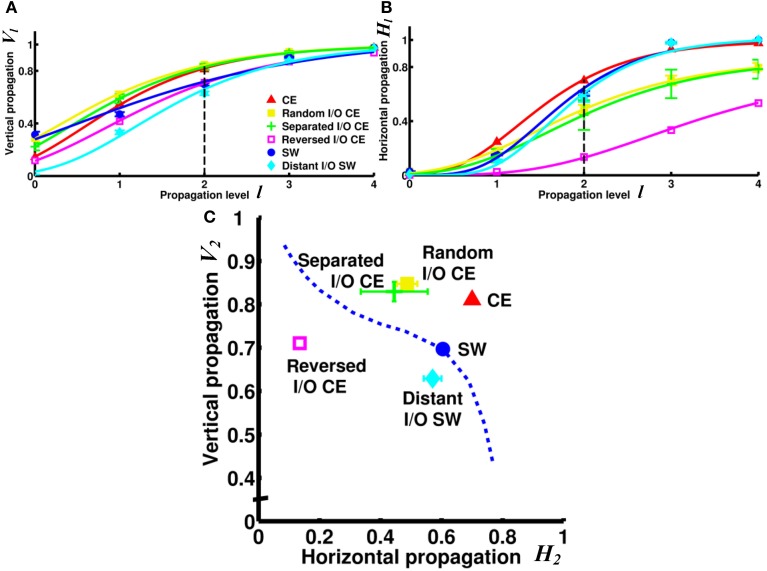
**Vertical and/or horizontal propagation become less efficient in CE and SW networks upon re-assignment of input and output nodes. (A)** The degree of vertical propagation decreases when we reverse the polarity of the CE network by interchanging the input and output neurons. In the SW network, the degree of vertical propagation also decreases in the distant I/O arrangement. **(B)** The degree of horizontal propagation of the CE network is reduced with all methods of reassignment (random, separated, and reversed). **(C)** On the V_2_ - H_2_ plot, the re-assigned networks are characterized by worse vertical and/or horizontal propagation. The reversed I/O CE network has the greatest reduction in both vertical and horizontal propagation. This result suggests that even when the network architecture remains intact, the efficiency of information propagation is highly dependent on the selection of input and output nodes, or the polarity of the network. Error bars indicate standard deviation calculated from 100 realizations.

Next, we asked a fundamental question: Under the same network architecture, is polarity crucial for the efficiency of information propagation? To address this question, we changed the polarity by selecting different nodes/neurons as input and output for the small-world and CE networks and investigated the impact of this on the degrees of vertical and horizontal propagation. In the preceding analyses (Figures 3–5), the input and output nodes in the SW network were selected randomly and each node type accounts for about 1/3 of the total. Consequently, input and output nodes are intermixed in the networks and a local cluster often contains nodes from both types. Here we assigned the input and output nodes differently so that the input and output nodes were located far away from each other and on opposite sides of the ring (see Methods). We also changed the polarity of the CE network by re-assigning the input and output neurons in the CE network in three different ways (see Methods). We compared the four networks (one small-world and three CE networks) and found that their channel connectivity matrices were now greatly changed (Figure 6). Further analysis of vertical and horizontal propagation revealed that the SW networks with distant I/O showed a significantly lower degree of vertical propagation than did the original SW networks (Figure 7). The result is easy to explain: in the original SW networks, each input node was likely to be in a local cluster containing one or several output nodes. Therefore, they were quickly connected by a large number of pathways that lead to highly efficient vertical propagation. In contrast, the SW networks with distant I/O had input and output nodes far away from each other. Therefore, the two types of nodes were connected via the long range projections and the number of pathways connecting each pair of input/output nodes was much smaller than those in the original SW networks.

Interestingly, the three re-arranged CE networks showed a very significant decrease in the degree of horizontal propagation compared to the original CE network. The CE networks with reversed I/O also exhibited a lower degree of vertical propagation (Figure 7). The results showed that the original CE network is highly optimized in terms of the efficiency of vertical and horizontal propagation. Re-designating input and output neurons resulted in overall lower propagation efficiency.

**Figure 8:**
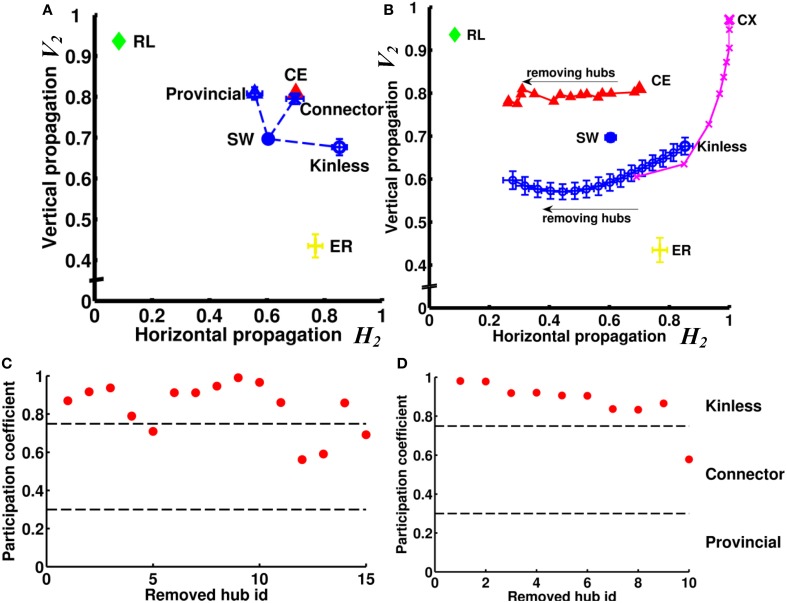
**Hub neurons/nodes play a crucial role in the efficiency of vertical and horizontal propagation. (A)** Without changing the numbers of nodes and links, we rearranged the links in an SW network to create 15 hub nodes. We found that creation of provincial hubs mainly improves vertical propagation. On the other hand, creation of kinless hubs improves horizontal propagation. Interestingly, by creating connector hubs endowed with both kinless and provincial properties, we can improve both vertical and horizontal propagation. **(B)** When we remove hub nodes one by one from an SW network endowed with kinless hub nodes, the efficiency of horizontal propagation is reduced. When we remove hub neurons one by one from the CE network, the change in propagation efficiency follows a similar trend, indicating that the hub neurons in the CE network are mainly of the kinless type. We also remove 10 hubs from the CX network. Note that the removals also result in significant changes in propagation efficiency but mainly in the vertical direction. Error bars indicate standard deviation calculated from 100 realizations. **(C)** By calculating participation coefficients for the 15 hub neurons, we find that most hub neurons in CE are kinless. Hub neurons are ranked by their connection numbers from high (left) to low (right) on the x-axis. **(D)** Same as in **(C)** but for the top 10 hubs in the CX network.

We next asked how the neural networks wire to achieve such high degrees of both vertical and horizontal propagation. We hypothesized that the “hub” neurons, i.e., neurons with connection numbers (in-degree + out-degree) much higher than the average of the network, play a critical role because they can easily propagate signals between different channels and/or enhance recurrent signal propagation within a channel due to the large number of inward and outward links. We first tested, using the small-world networks as model networks, how different types of hub nodes influence the vertical and horizontal propagation. We tested three types of hub: provincial, connector, and kinless. We replaced 15 nodes in the small-world network with 15 hub nodes of a specific type while keeping the total number of nodes and links constant (see Supplementary Method) (Figure 8A). We found that the provincial hubs, characterized by restricting all their connections to the same module, significantly improved the degree of vertical propagation while the horizontal propagation only slightly decreased. (*V*^SW−provincial^_2_ = 0.81, *H*^SW−provincial^_2_ = 0.56, *V*^SW−original^_2_ = 0.70, *H*^SW−original^_2_ = 0.60). In contrast, the kinless hubs, characterized by uniform projections to the entire network, significantly increased the degree of horizontal propagation while not significantly changing the vertical propagation (*V*^SW−kinless^_2_ = 0.68, *H*^SW−kinless^_2_ = 0.85). The connector hubs, which project partially outside and partially within the module that the hub node resides in, improved both the vertical and the horizontal propagation. (*V*^SW−connector^_2_ = 0.80, *H*^SW−connector^_2_ = 0.70). Next, we tested the CE network by removing 15 hub neurons from the network individually (Figure 8B). The 15 hub neurons were determined by finding the 15 neurons possessing the largest number of connections (sum of in-degree and out-degree). We discovered that the removals resulted in decreasing the degree of horizontal propagation but not vertical propagation. The result suggested that hub neurons in the CE network are mainly of the kinless type. Indeed, when we removed hub nodes from a small-world network endowed with kinless hub nodes, we reproduced this effect (Figure 8B). To further verify the type of the hub neurons in the CE neural network, we evaluated the modularity of the network based on the algorithm proposed in (Leicht and Newman, 2008; Rubinov and Sporns, 2010). By analyzing the participation coefficients of the connections of each hub neuron, we found that most of the hub neurons are indeed of the kinless type (Figure 8C). Interestingly, hubs in the CX network exert different influences than do those in the CE network. By removing 10 hubs from a CX network, we observed a rapid decrease of degree of vertical propagation with the first few removals and then a decrease in the degree of horizontal propagation with later removals (Figure 8B). Interestingly, analyzing the participation coefficients of the 10 hubs showed that they were kinless hubs (Figure 8D), not theoretically expected to greatly affect vertical propagation. To find out why removing them reduced the degree of vertical propagation, we carefully inspected these 10 hubs and discovered that they shared similar connectivity patterns. Two of the 10 hubs were input neurons that had divergent projections to all output neurons. The other eight hubs were interneurons that received information from all input neurons and originated convergent projections to a few output neurons. Therefore, these hubs form a redundant circuit in terms of horizontal propagation. If we remove several hubs from the network, the rest of the hub neurons are still able to quickly propagate information from any input neuron to all output neurons. As a result, the degree of horizontal propagation remains high. In contrast, these hub neurons play an important role in establishing recurrent circuits; hence, each hub removal results in a significant decrease in the number of pathways between input neurons and output neurons, leading to a reduced degree of vertical propagation.

**Figure 9:**
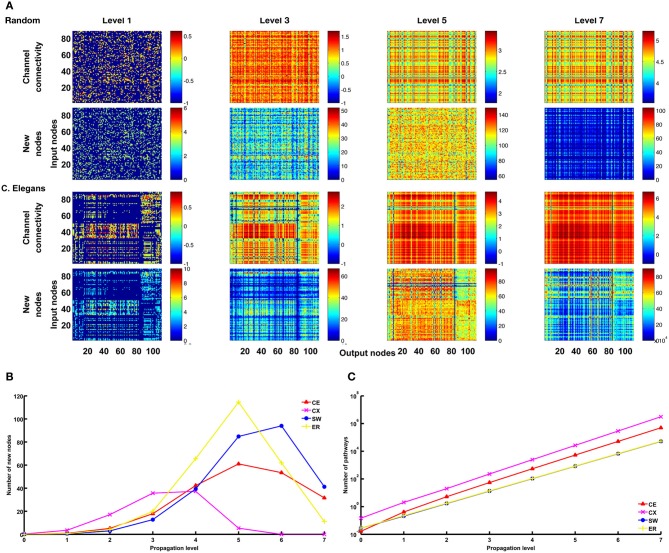
**Large numbers of pathways at higher propagation levels are mainly contributed by recurrent connections. (A)** For the ER and CE networks, we plot matrices for channel connectivity (rows 1 and 3) and numbers of new nodes (rows 2 and 4). Note that when propagation level is higher than 5, the numbers of new nodes start to decrease while the numbers of pathways as indicated by the channel connectivity matrices still increase rapidly. **(B)** The mean number (averaged over all channels) of new nodes initially increases with propagation level but then drops rapidly for CE, CX, SW, and ER networks. **(C)** The mean number of pathways grow exponentially with propagation level for all four networks. These trends are expected for recurrent networks. The data of SW and ER networks shown in **(B)** and **(C)** were averaged over 100 realizations for each network.

We mentioned earlier that a large number of pathways between an input and an output node at high propagation levels indicates the involvement of strongly recurrent connections. The statement can be verified by examining the number of new nodes included in the pathways at each level (Figure 9A). We found that, for the CE, CX, SW, and ER networks, as propagation level increases, the number of new nodes drops rapidly while the number of pathways increases exponentially (Figures 9B,C). This is consistent with what is expected for a highly recurrent network.

## Discussion

In the present study, we proposed a novel method to quantify the architecture of a network in terms of numbers of pathways between input and output nodes at different propagation levels. Our analysis was based on two critical aspects: (1) the polarity of the network, and (2) the contribution of higher level pathways. Considering that a large number of pathways indicates the involvement of a large number of nodes and connections, we interpret the number of pathways as the amount of computation that may occur between the input and output nodes.

We argue that a neural network should not simply relay signals from input nodes to output nodes, but should rather perform a complex computation via two fundamental processes: (1) Transformation: After entering an input node, a signal is transformed, or computed, by propagating through several nodes and pathways before leaving at a specific output node. (2) Combination: New information can be generated by combining signals from different input nodes. We identified the transformation process with vertical propagation because it indicates the amount of computation that may occur between a specific input node and output node. We identified the combination process with horizontal propagation because it measures how many input nodes that each output node receives signals from.

**Figure 10:**
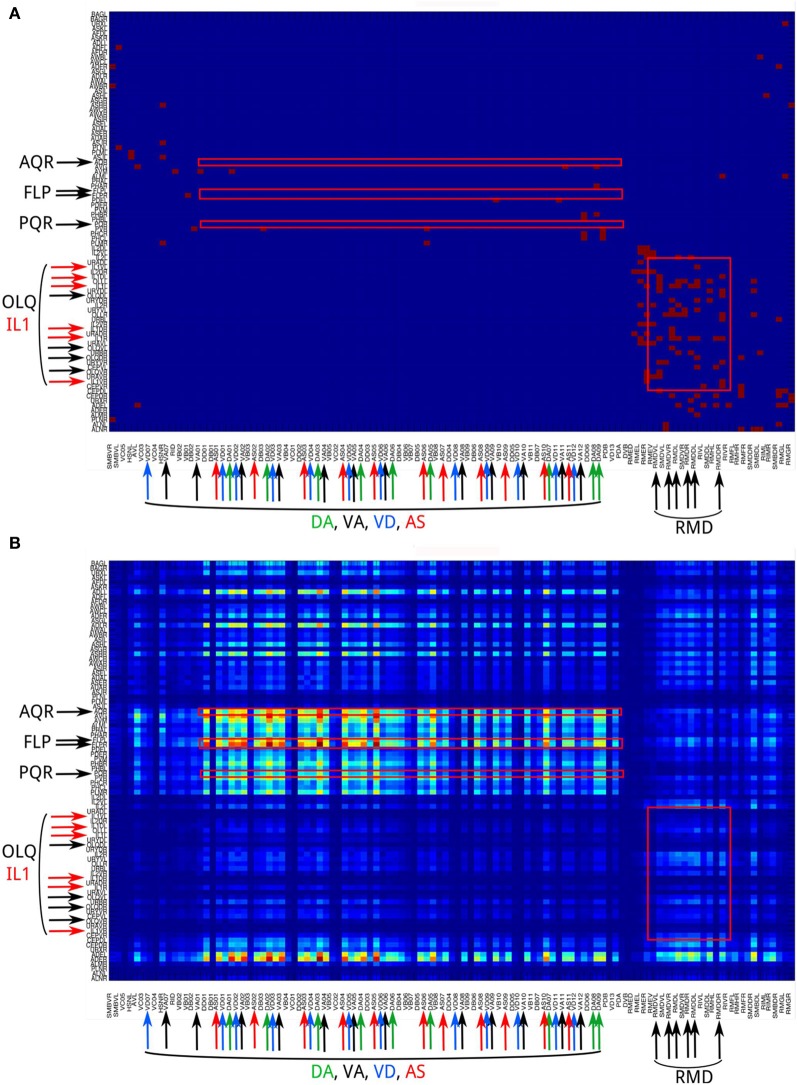
**The channel connectivity matrix of the *C. elegans* neural network at propagation levels 0 (A) and 3 (B)**. Each input or output neuron is labeled with its anatomical name. Neurons on the y-axis (input neurons) are displayed in reverse order compared to the matrix shown in Figure 2. We also labeled the neuron groups mentioned in the Discussion using text with larger fonts and color-matched arrows. Red squares indicate several regions of interest that represent the connections (1) AQR and PQR neurons to DA, VA, VD, and AS neurons; (2) FLP neurons to DA, VA, VD, and AS neurons; and (3) OLQ and IL1 neurons to RMD neurons. **(A)** At level 0, only OLQ/IL1 and RMD are heavily connected. These connections mediate the head-withdrawal reflex elicited when a worm is touched with an eyelash on either the dorsal or ventral sides of its nose. **(B)** At level 3, the sensory neuron groups AQR, PQR, and FLP are strongly connected to the motor neuron groups DA, VA, VD, and AS. In contrast, the sensory neuron groups OLQ and ILQ, heavily connected to RMD at level 0, are only weakly connected at level 3. To visualize the difference between strong and weak connections, here we plot the matrix using number of pathways [*m*(*i, j*), see Methods] directly rather than taking the logarithmic values [*M*(*i, j*), see Methods].

We found that compared with classic small-world networks, neural networks are more efficient in vertical and horizontal information propagation. Our result leads to several novel conclusions:

1. The efficiency of information propagation is sensitive to network polarity or the assignment of input and output nodes. Even for networks of the same architecture, if we assign the input and output nodes differently, the efficiency of vertical and/or horizontal propagation changes significantly. This result highlights the importance of considering the network architecture in conjunction with network polarity when analyzing a neural network.
2. Neural networks show a distinct difference in channel connectivity patterns between level 0 (direct links) and level 1 (indirect links via single intermediate neurons). This is evident from the channel connectivity matrices (Figure 3) as well as from the degree of vertical propagation (Figure 5A). These characteristics suggest that direct and higher-level links support distinct functions. Although some signals may quickly and directly propagate from input nodes to output nodes, a different connectivity pattern emerges when we consider the higher-level connections, which may have a different functionality. Indeed, nervous systems are often endowed with direct or express connections by which the input signals can trigger reflexive or automatic responses, while the same signals may also propagate through multiple intermediate neurons in highly recurrent circuits that produce complex and cognitively higher-level behaviors. For example, *C. elegans* exhibits a head-withdrawal reflex in which the worm interrupts the normal pattern of foraging and executes an aversive head-withdrawal response when touched by an eyelash on either the dorsal or ventral sides of the head. This simple reflex is mediated by mechanosensory neurons (OLQ and IL1) and the RMD motor neurons (Hart et al., 1995; Riddle et al., 1997) which form many direct connections at the propagation level 0 (Figure 10A) but have relatively low pathway numbers at higher levels (Figure 10B). In contrast, there is a large number of pathways between the oxygen sensory neurons (AQR and PQR) and the locomotion neurons (DA, VA, VD, and AS) at higher propagation levels (Figure 10B). Several studies have revealed that the worms change their social feeding behavior when the oxygen concentration changes (Coates and de Bono, 2002; Cheung et al., 2005; Busch et al., 2012). We also observed a significantly higher number of pathways between FLP sensory neurons and locomotion neurons (DA, VA, VD, and AS) at level 3 (Figure 10B). FLP neurons are involved in multiple functions, including the harsh noise touch response, the gentle nose touch response, and the heat avoidance response (Goodman, 2006; Chatzigeorgiou and Schafer, 2011; Liu et al., 2012).
3. Our finding of kinless hub neurons in the CE network has important implications for our understanding of how neural networks are constructed. We often compare neural networks with small-world networks of similar clustering coefficient and mean shortest path length. However, our analysis suggests the possibility that a neural network is formed from several base network modules (or clusters) to which a small number of kinless hub neurons is added, mainly to improve horizontal propagation efficiency.

We demonstrated in Figure 9 that large values in the channel connectivity matrix at high propagation levels are associated with strongly recurrent pathways. So why is this association significant? First, almost all neural circuits are recurrent. Even for the circuits such as those in retina and olfactory bulb, in which signals are propagated in a channel-like feedforward fashion, there are always lateral interneurons that propagate information back and forth between channels. Second, various biological neural network models have demonstrated that recurrent circuits perform variety of information integration and computation which support functions including oscillation, working memory, perceptual discrimination, etc. (Brunel, 2000; Compte et al., 2000; Wang, 2002; Machens et al., 2005; Lo and Wang, 2006). Although a convergent feedforward circuit can also integrate and transform information, the signals come and go. In contrast, the recurrent pathways are able to retain information and integrate information over time. This gives the recurrent pathways an extra (temporal) dimension to process information comparing to feedforward pathways. Based on this consideration, the input and output neurons which are connected by large pathway numbers at high propagation levels can be treated as indicators of information processing hotspot due to the involvement of strongly recurrent circuit.

We note that sometimes signals propagate not only from input to output neurons, but also from output back to input neurons. For example, movement-related neurons may send efferent copies back to sensory-related neurons. To analyze the backward propagation, one can simply reverse the assignment of input and output nodes in a network as we did in the reverse I/O CE network (Figures 6, 7). Examining the result, we found that the backward propagation in the CE network has a slightly smaller degree of vertical propagation and a much smaller degree of horizontal propagation than those of the forward propagation. The result implies that, the feedback propagation from the motor neurons acts on fewer sensory neurons and requires more time to take effect than the sensory neurons do on motor neurons in the forward propagation. Although the result cannot tell us the exact functions of the forward and backward propagations and why their efficiencies are different, the result may stimulate further experimental investigations.

We emphasize that the assignment of input and output nodes is flexible in our algorithm and is dependent on what the users want to analyze. In fact, changing assignment of input and output nodes as discussed above has interesting applications. One can study the efficiency of information propagation between any two neuron populations of interest in a network by assigning one population as input nodes and the other as output nodes. Furthermore, for a neural network with unclear polarity, i.e., no input and output neuron identified, one can identify the potential polarity by assigning different neural populations in the network as input and output nodes and analyzing the efficiency of information propagation for the different assignments.

**Figure 11:**
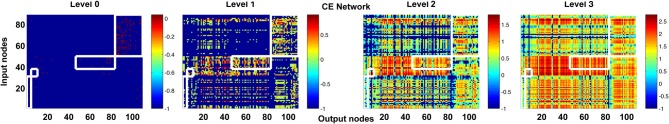
**Modularity analysis of level 0 connectivity does not fully reveal the information about high-level connectivity**. We performed modularity analysis of the adjacency matrix (level 0 connections) of the CE network and indicate the input and output neurons that are classified into the same modules by white boxes in the channel connectivity matrices. At level 3, many hotspots (strongly connected neurons, as indicated by hot colors) appear outside the white boxes and some input and output neurons in the white boxes become weakly connected. The result suggests that high-level matrices do provide new information that is not available in the modularity analysis of the adjacency matrix.

One may argue that our analysis of high-propagation-level matrices is not necessary because if we perform a modularity analysis of the adjacency matrix, the pairs of input and output nodes that are only heavily connected at higher levels will be clustered into the same modules. Therefore, in spite of no direction connection at level 0, the high-level connectivity of these input and output nodes can still be captured by simple modularity analysis of the adjacency matrix. To address this concern, we performed the modularity analysis of the *C. elegans* network using the algorithm proposed in (Leicht and Newman, 2008; Rubinov and Sporns, 2010). We found that a large portion of the input and output neurons which are heavily connected at higher levels are actually not clustered into the same modules in the adjacency matrix (Figure 11).

Our analysis does not distinguish between excitatory and inhibitory neurons in a network. One may argue that our analysis over-estimates the number of pathways between neurons because two signals can cancel each other if one arrives from an excitatory pathway and the other from an inhibitory pathway. However, an increasing number of empirical and theoretical studies have shown that the excitatory and inhibitory signals do not simply cancel each other. These signals form a balanced state which enriches the dynamics of the network and provides modulatory signals for the networks (Chance et al., 2002; Abbott and Chance, 2005; Mariño et al., 2005; Berg et al., 2007; Siegle and Moore, 2011; Yizhar et al., 2011; Wang et al., 2013). In addition to dynamical balancing, inhibitory neurons have also been suggested to participate in various other functions such as neuron competition and oscillation, in which inhibitory neurons convey information. Therefore, we included all nodes regardless their excitatory or inhibitory properties to ensure that we are not missing any possible pathways that may participate in neural computation. One may tend to use negative values in the adjacency matrix for inhibitory synapses. However, due to the diverse functions that inhibitory neurons potentially participate in as mentioned above, we suggest that unless a strong signal-blocking effect are observed empirically for specific inhibitory synapses, we should not use negative values for the corresponding elements in the matrix.

Several issues remain to be addressed in follow-up studies. First, how do vertical and horizontal propagation scale with network size? Although the response time to simple stimuli is generally faster for smaller animals due to the shorter transmission pathways in the sensory-motor nervous system, cognitive ability is generally higher in animals with larger brains or larger numbers of neurons (Deaner et al., 2007; Sol et al., 2008; Isler and van Schaik, 2009; Rushton and Ankney, 2009). Our analysis, based on the number of pathways between paired input and output nodes at higher propagation levels, can potentially be used to measure the complexity of information processing. Therefore, our method may provide insights into correlations between cognitive ability and network properties. Second, connections in neural networks are weighted, meaning that the strengths of synapses vary significantly across a neural network. Although measuring weights for all synapses in a neural circuit (in particular *in vivo*) remains technically challenging today, this information will gradually become available as technology advances. We will need to incorporate connection weights into our algorithm to gain a better picture of information propagation in networks. Once the information about synaptic weight is available, it will be very interesting to investigate the effect of synaptic plasticity on the information propagation using our method. For example, the influence of short-term facilitation (or depression) can be simulated by increasing (or reducing) the value of the corresponding element in the level 0 matrix. Furthermore, functional networks can be very different from the anatomical networks due to the information gating arisen from synaptic plasticity or local inhibition at key connections. The influence of the information gating on high-level propagation can also be analyzed using our method. Although changing the values of a few elements (connections) may not produce much impact on the connectivity matrix at level 0, the small changes may accumulate and eventually alter the higher level matrices in a significant way. Moreover, new functional predictions may be generated by our analysis if we remove neurons or pathways one-by-one in the adjacency matrix and see how each removal changes the high-level connectivity. Third, our analysis demonstrates the importance of hubs in gaining high degrees of vertical and/or horizontal propagation. However, comparing the hub neurons in CE and CX neural networks revealed that the same type of hub can have different influences on information propagation in different networks. Therefore, the existing classification of hubs (provincial, connector, kinless) may not be sufficient for the analysis of information propagation. We need to identify exactly which properties of hubs individually affect vertical or horizontal propagation and develop a new classification of hub neurons. Fourth, our analysis demonstrated that at high propagation levels the patterns of connectivity between input and output nodes are very different from those formed by direct connections (level 0). Therefore, it may be worthwhile to modify and apply other analytical methods such as modularity and motif to the “new” networks formed by high-level pathways. In this way, we can avoid interference from the direct links while measuring the properties associated with connections through multiple and recurrent links, which may be more relevant to high-level neural circuit functions.

### Conflict of interest statement

The authors declare that the research was conducted in the absence of any commercial or financial relationships that could be construed as a potential conflict of interest.
