# Supplementary material for: Polarity-specific high-level information propagation in neural networks
Source: Front Neuroinform. 2014 Mar 17;8:27. doi: 10.3389/fninf.2014.00027 (PMC3955877; doi:10.3389/fninf.2014.00027)
Supplement: Supplementary file 1 [file Presentation1.PDF]

Supplementary Material

Polarity-specific high-level information propagation in neural networks

Yen-Nan Lin[1], Po-Yen Chang[1], Pao-Yueh Hsiao[1] and Chung-Chuan Lo[1, 2] *

[1]Institute of Systems Neuroscience, National Tsing Hua University, Hsinchu 30013, Taiwan.

[2]Department of Life Science, National Tsing Hua University, Hsinchu 30013, Taiwan.

*** Correspondence:** Chung-Chuan Lo, Institute of Systems Neuroscience, National Tsing Hua University, No. 101, Section 2, Kuang-Fu Road, Hsinchu, 30013, Taiwan
cclo@mx.nthu.edu.tw

1. Supplementary Method

Drosophila partial central complex (CX) neural network

The *Drosophila* partial CX neural network was derived from a previously published database(1). The authors used the adapted mosaic analysis with a repressible cell marker (MARCM) labeling technique (2) to reconstructed 659 neurons that innervate the protocerebral bridge (PB), a neuropil that forms a major part of the central complex along with other neuropils including the fan-shaped body (FB), the ellipsoid body (EB), the noduli (NO) etc. The authors further divided each of the central complex neuropils and other related neuropils into multiple sub-regions based on the glomerular structures observed in these neuropils. Next, the authors registered the sub-regions that each neuron innervates, classified the polarity (axons or dendrites) and then identified 194 unique innervation patterns from the 659 neurons. The redundancy is likely to arise from the experimental technique in which same neurons may be repeatedly recorded from different samples. In the present study we build the partial CX network based on the 194 innervation patterns. To generate the network, or the connectivity matrix, from the innervation patterns, we assumed that if a neuron innervates a subregion with an axon and another neuron innervates the same subregion with a dendrite, these two neurons are connected. The assumption can be justified by considering the small average size ($14.2\mu m$) of the sub-regions. Furthermore, most neurons innervate sub-regions with dense glomerular structures that cover the entire sub-regions (1). Therefore, it is highly probable that dendrites and axons from different neurons that innervate the same sub-region form synaptic contacts.

Creating hubs in small-world networks

In addition to classifying types of hubs and studying their contribution in the information propagation in CE network, we also investigated how different types of hubs influence the vertical and horizontal propagation in SW networks by manually introducing hub nodes. To this end, we replaced 15 randomly selected nodes by hubs using the following procedure. Assuming the SW network consists of N nodes with a mean of k connections per node. We first constructed a degree distribution which was used to determine the number of links for the 15 hubs. The degree distribution followed an exponential decay as the type of distribution has been observed in several neural networks including *C. Elegans* (3, 4). Specifically, we used the following shifted probability density function of exponential distribution:

$$f(x,\lambda)=\begin{cases}\lambda\exp^{-\lambda(x-1)} & \text{when } x\geq 1\\ 0 & \text{when } x<1\end{cases} \quad [1]$$

The distribution was shifted by one in order to prevent from the creation of zero-connection nodes. We set $\lambda=\frac{1}{k-1}$ so that the resulting mean of the distribution eqauled k exactly. Next, we drew N numbers randomly from the shifted exponential distribution, picked the largest 15 numbers and used than as the number of links for the 15 hub nodes we selected.

To maintain the total number of links in the networks, after we replace 15 nodes with hubs, we uniformly decreased the degree of non-hub nodes. The new mean number of links is given by:

$$\frac{k\times N-\sum_{i}^{15}k^{hub}{}_{i}}{N-15}, \quad [2]$$

where $k^{hub}{}_i$ is the number of links of the i-th hub node.

To test the effects of the hub class (provincial, connector or kinless) in the vertical and horizontal propagations, in each network realization, we replaced 15 randomly selected nodes with hubs of a specific class. However, the class of a hub is defined by its relative distribution of links between modules, while modules in a network can only be identified after all links have been established. So it is difficulty to specify the class of a hub when we create it as the information about modules is not available at the time of creation. We addressed the issue by adopting the following three mechanisms for the link arrangement when we created hubs. We found that the three mechanisms give rise to three different classes of hubs.

1. We connected all of the $k^{hub}{}_i$ links of a hub with its neighboring nodes in the ring lattice. To produce a high in-degree for the hub, each connected neighbor randomly rewired one of its existing links and projected the link back to the hub if such a feedback link was not already exist. We found that using this procedure, 70% of the created hubs were provincial.
2. We connected all of the $k^{hub}{}_i$ links of a hub with randomly selected nodes. To produce a high in-degree for the hub, we randomly selected the same number of nodes and rewired one of its existing links by projecting the link back to the hub if such a feedback link was not already exist. Using this mechanism, 99% of the created hubs were kinless.
3. We connected 80% of the $k^{hub}{}_i$ links of a hub with neighboring nodes and connect the rest of the links with randomly selected nodes. To produce a high in-degree for the hub, each of the neighboring connected nodes projected one of its randomly selected links back to the hub if the feedback link was not exist. We next randomly selected 0.2* $k^{hub}{}_i$ number of nodes and projected one of each node's links back to the hub if the feedback link is not already exist. We found that using this mechanism, 97% of the created hubs were connector.

2. References

Amaral, L. a. N., Scala, A., Barthélémy, M., and Stanley, H. E. (2000). Classes of Small-World Networks. *PNAS* 97, 11149–11152. doi:10.1073/pnas.200327197.

Chiang, A.-S., Lin, C.-Y., Chuang, C.-C., Chang, H.-M., Hsieh, C.-H., Yeh, C.-W., Shih, C.-T., Wu, J.-J., Wang, G.-T., Chen, Y.-C., et al. (2011). Three-Dimensional Reconstruction of Brain-wide Wiring Networks in Drosophila at Single-Cell Resolution. *Current Biology* 21, 1–11. doi:10.1016/j.cub.2010.11.056.

Lin, C.-Y., Chuang, C.-C., Hua, T.-E., Chen, C.-C., Dickson, B. J., Greenspan, R. J., and Chiang, A.-S. (2013). A Comprehensive Wiring Diagram of the Protocerebral Bridge for Visual Information Processing in the Drosophila Brain. *Cell Reports* 3, 1739–1753. doi:10.1016/j.celrep.2013.04.022.

Modha, D. S., and Singh, R. (2010). Network architecture of the long-distance pathways in the macaque brain. *Proceedings of the National Academy of Sciences* 107, 13485 –13490. doi:10.1073/pnas.1008054107.
